# Period of Infectiousness of Contagious Diseases

**Published:** 1893-09

**Authors:** 


### PERIOD OF INFECTIOUSNESS OF CONTAGIOUS DISEASES.

Small-Pox.—Six weeks from the commencement of the disease if every scab has fallen off.

Chicken-Pox.—Three weeks from the commencement of the disease, if every scab has fallen off.

Scarlet Fever.—Six weeks from the commencement of the disease, if the peeling has ceased and there is no sore nose.

Diphtheria.—Six weeks from the commencement of the disease, if sore throat and other signs of the disease have disappeared.

Measles.—Three weeks from the commencement of the disease, if all rash and the cough have ceased.

Mumps.—Three weeks from the commencement of the disease, if all swelling has subsided.

Typhus.—Four weeks from the commencement of the disease, if strength is re-established.

Whooping Cough.—Six weeks from the commencement of the disease, if all cough has ceased,

Typhoid.—Six weeks from the commencement of the disease, if strength is re-established.

Under judicious treatment the period of infectiousness may be considerably shortened, but *no child suffering as above should be admitted to any school afte1 a shorter period of absence, and should be provided with a medical certificate* that he or she is not liable to communicate the disease.

Length of quarantine.—Teachers or children *who have been exposed* to infection from any of the following diseases may safely be re-admitted to the school, if they remain in good health (and have taken proper means for disinfection) after the following periods of quarantine :

Diphtheria, 12 days ; scarlet fever, 14 days ; small pox, 18 days ; measles, 18 days ; chicken pox, 18 days ; mumps, 24 days ; whooping cough, 21 days.

Adults may be re-admitted immediately, if they disinfect their clothes and persons.—*Regulations, State Board of Health of Pennsylvania.*

